# The prevalence of lymphoedema in women who attended an information and exercise class to reduce the risk of breast cancer-related upper limb lymphoedema

**DOI:** 10.1186/s40064-015-1629-8

**Published:** 2016-01-07

**Authors:** E. Jeffs, A. Purushotham

**Affiliations:** Florence Nightingale Faculty of Nursing and Midwifery, King’s College London, James Clerk Maxwell Building, Waterloo Campus, 57 Waterloo Road, London, SE1 8WA UK; Guy’s and St Thomas NHS Foundation Trust, Guy’s Hospital, 3rd Floor Bermondsey Wing, Great Maze Pond, London, SE1 9RT UK

**Keywords:** Breast cancer, Lymphoedema, Exercise, Prevalence, Risk reduction

## Abstract

Breast cancer-related upper limb lymphoedema (BCRL) affects approximately 20 % of women undergoing axillary intervention. Women who attended a “reducing your risk of lymphoedema” class, including exercise instruction, anecdotally reported positive BCRL outcomes. The aim of this study was to examine BCRL outcomes and perceived benefit for attendees at a “reducing your risk of lymphoedema” class between 2000 and 2005. A cross-sectional study was conducted in two parts: (1) self-report questionnaire regarding lymphoedema status and benefit received from class and exercise programme; (2) clinical evaluation and objective measurement to confirm BCRL. 46 women completed questionnaires; 40 continued to clinical evaluation and objective measurement. BCRL prevalence defined as ≥10 % excess limb volume was only 5 %, although clinician judgement identified 23 % with arm lymphoedema and 8 % with lymphoedema limited to the hand. Clinician judgement correlated highly with patient self-report (Kappa = 0.833, *p* = 0.000). All women found the class beneficial, reporting increased confidence to return to normal life and a wide range of activities/exercise. We conclude that prevalence of BCRL should be determined by both clinical judgement and objective measurement to avoid underestimation. The benefit of group education with a lymphoedema expert and of exercise instruction should be further explored, and the potential for exercise to reduce BCRL prevalence should be examined.

## Background

Lymphoedema is a common consequence of breast cancer treatment affecting approximately 20 % of women undergoing axillary intervention, with the majority developing swelling within 2 years (Disipio et al. 2013). The impact of breast cancer-related upper limb lymphoedema (BCRL) on the survivor is significant, including physical changes, impairment to function and daily life activities, challenges for work, social and leisure activities, and financial implications (Shih et al. 2009; Carter 1997; Fu 2008; Radina 2009). BCRL causes considerable psychological distress (Fu et al. 2013), and can alter body image and act as a visible reminder of breast cancer and its treatment (Vassard et al. 2010).

Women in the UK at risk of developing BCRL are routinely offered verbal and written advice regarding care and use of the arm (e.g. leaflets from Breast Cancer Care, Lymphoedema Support Network, Macmillan Cancer Support), with many services offering additional group education sessions. As part of a review of breast cancer care at our hospital we wished to explore the potential impact on BCRL outcome of a proactive approach to advice and education regarding exercise.

The role of exercise and movement to influence lymphatic and venous drainage is well recognised (Foldi and Foldi 2012; Lymphoedema Framework 2006; International Society of Lymphology 2013). It is now accepted that appropriately performed exercise does not cause or exacerbate BCRL (Cheema et al. 2014; Kwan et al. 2011; Stuiver et al. 2015). However, for many years women with and at risk of developing BCRL were advised to avoid any strenuous activity or exercise (Cemal et al. 2011; Lee et al. 2009; Nielsen et al. 2008), and many UK women still report precautionary behaviours; these include limiting use of their affected arm, and a desire for greater guidance regarding how to safely return to pre-treatment exercise and activity levels (anecdotal evidence).

We decided to review the outcomes of a group of women who had attended a “reducing your risk of lymphoedema” education and advice class (taught by author EJ) between 2000 and 2005; to our knowledge, none of the women subsequently reported development of BCRL. The class had included teaching of a simple exercise programme which had originally been developed by the author (EJ) in 2000 at the request of women with BCRL who wanted to know how to safely return to pre-treatment exercise; they wished to recommence activities such as gym, playing tennis, golf, without triggering or exacerbating BCRL. So we wanted to know whether the “at risk” group had in fact achieved better lymphoedema outcomes than might be expected in such a group. Also, we knew that some women with established BCRL (n = 21) had used the bespoke exercise programme added to usual care and demonstrated a small but clinically and statistically significant greater reduction in excess limb volume (ELV) when compared to usual care alone (Jeffs and Wiseman 2013).

The primary objective of this study was to determine prevalence of clinically detectable BCRL in women who attended a “reducing your risk of lymphoedema” class between 2000 and 2005. In addition, we intended to observe how ELV and participant perception of BCRL compared with clinician assessment, so secondary objectives included determining the number of women with ELV ≥10 % and patient-perceived BCRL, and the agreement between all three methods. Finally, we wanted to know the patient-perceived benefit of attending the “reducing your risk of lymphoedema” class.

## Participants and methods

The cross-sectional study was designed in two parts:

1. Self-report questionnaire to ascertain patient report of BCRL, and perceived benefit of attending the “reducing your risk of lymphoedema” class and undertaking the exercise programme;
2. Clinical assessment to confirm presence of BCRL and determine percentage ELV.

### Participants

The participants were women who had attended a “reducing your risk of lymphoedema” class, taught by the author (EJ) at a London breast cancer support charity between 2000 and 2005 (Jeffs 2006). The 1½ h class consisted of two parts: (1) explanation of BCRL, risk, and measures that may reduce risk of developing BCRL; (2) demonstration and practice of a simple 10–15 min exercise routine for daily home use. The exercise routine consisted of a proximal to distal sequence of deep breathing and gravity-resistive isotonic arm exercises (flexibility and strengthening) designed to stimulate lymphatic and venous return. The class encouraged a return to pre-treatment exercise and activities at a time when women receiving breast cancer treatment in the UK were routinely advised not to lift more than 3 lb weight.

**Fig. 1:**
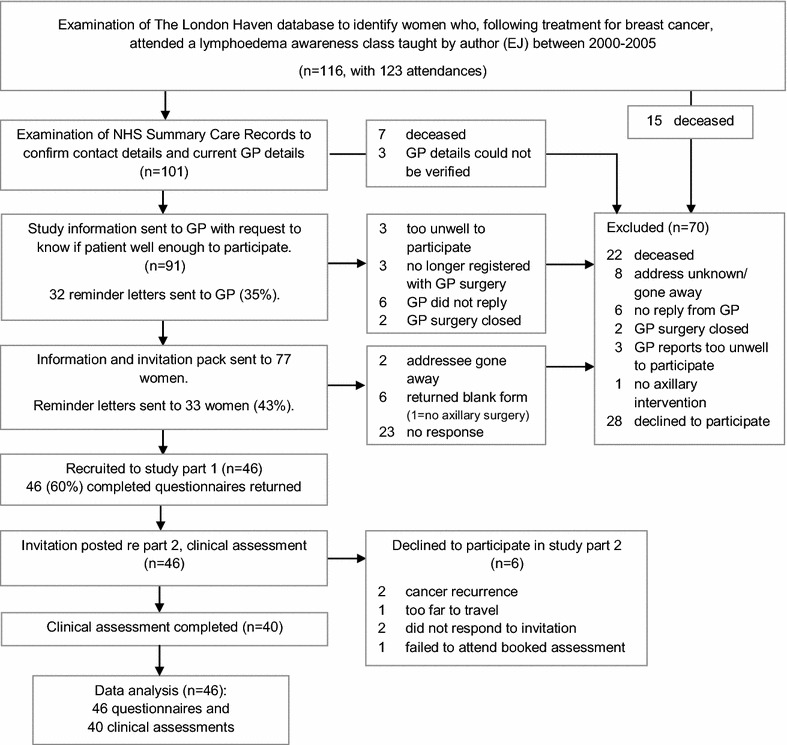
Participant flow through trial

Records of the London breast cancer support charity were examined, identifying attendees at a total of 13 lymphoedema awareness classes (see Fig. 1). Women were excluded if they could not be contacted, were deceased, not well enough to participate, or had attended a class not taught by the author (EJ). As it was likely that, unbeknownst to the charity, some women were deceased or suffering from progressive disease (Cancer Research UK 2015), we requested individual primary care providers (GPs) to confirm whether their patient was well enough to participate; 83 GPs (91 %) responded (see Fig. 1).

### Data collection

In 2012, questionnaires were sent to 77 women with an explanatory letter, study information sheet, and stamped addressed envelope to return their completed questionnaire. One reminder letter was sent 6 weeks later with another copy of questionnaire and study information.

The questionnaire contained 27 items addressing three main areas: (1) breast cancer treatment; (2) development of swelling and associated symptoms; (3) views on “reducing your risk of lymphoedema” class and perceived benefit of exercise programme. It combined tick boxes with free text and additional space for further comments. Receipt of completed questionnaire was considered consent to part one of the study.

Each woman who returned a completed questionnaire was invited to attend for clinical assessment (study part two). One reminder letter was sent to those who did not respond or failed to attend a booked appointment.

Clinical assessment to determine the presence of BCRL followed the assessment method used in the author’s (EJ) clinical practice and previous research (Jeffs and Wiseman 2013), addressing:

- baseline demographics: age, weight, height, ethnicity, occupation, activity level;
- medical history: participant self-report of breast cancer treatment, current health status, onset and development of swelling, precipitating factors, lymphoedema precautions taken;
- clinical examination of both limbs to determine presence of oedema;
- participant perception of current arm symptoms: swelling, ache, heaviness, pain, numbness, tingling;
- clinical measurements:
  - limb volume, by Perometry, an infrared measuring device (Lee et al. 2011);
  - shoulder extension and abduction, using goniometry (Jeffs and Wiseman 2013; Valentine and Lewis 2006);
  - quality of life using LYMQOL, a lymphoedema-specific arm questionnaire (Keeley et al. 2010);
  - self-report arm and hand function, using QuickDASH (Kennedy et al. 2013).

### Diagnosing lymphoedema

For the purpose of this study, the presence of BCRL was determined by three methods used in incidence and prevalence studies (Armer and Stewart 2005; Disipio et al. 2013; Hayes et al. 2008a):

1. clinician judgement, with the presence of one or more symptom of oedema regardless of severity: decreased visibility of veins, increased thickness of skin and subcutis, fullness of tissues or smoothing of natural limb contours, pitting oedema (Stanton et al. 2006);
2. at least 10 % ELV, as measured by the Perometer;
3. patient self-report.

### Analysis

Data were analysed to determine participant homogeneity and associations for diagnosis of lymphoedema by three methods: (1) clinical judgement, (2) objective limb volume measurement, (3) patient report. Sensitivity and specificity were calculated for method (1) versus (2) and (1) versus (3), with kappa statistics calculated for all 3 methods as well as for each pair (Landis and Koch 1977).

Statistical analysis was carried out using SPSS version 20 (IBM, USA), with significance set at *p* < 0.05.

### Ethical issues

Approval was obtained from the National Research Ethics Service (NRES Committee London-Dulwich) and sponsoring hospital’s Research and Development department prior to commencement of the study. The study was conducted in compliance with the study protocol, ethical standards laid down in the 1964 Declaration of Helsinki and Good Clinical Practice guidelines for research. All participants gave informed written consent prior to inclusion in the study.

## Results

Forty-six women (60 %) completed questionnaires and 40 women (52 %) underwent a clinical assessment, a conversion rate of 87 %. Demographic and clinical characteristics for the 40 women (study part two) are shown in Tables 1 and 2. The only significant difference between women with and without clinician-determined lymphoedema were ELV and duration of lymphoedema.

**Table 1:** Demographic characteristics of total group and according to presence of clinician-determined BCRL

Characteristic	Total group (N = 40)	Lymphoedema present (N = 12)	No current swelling (N = 28)
Age, years (median, LQ, UQ)	62 (55, 66)	62 (55, 68)	63 (55, 66)
Ethnicity, N (%)			
White British/White Other	35 (88)	9 (75)	26 (93)
Black and minority ethnic	5 (13)	3 (25)	2 (7)
Employment, N (%)			
Professional/managerial	10 (25)	3 (25)	7 (25)
Clerical/service/administration	7 (18)	3 (25)	4 (14)
Skilled trade	2 (5)	1 (8)	1 (4)
Manual	1 (3)	0	1 (4)
Homemaker	2 (5)	0	2 (7)
Retired	16 (40)	5 (42)	11 (39)
Unemployed	1 (3)	0	1 (4)
Missing	1 (3)	0	1 (4)
BMI (median, LQ, UQ)	26.05 (23.27, 27.95)	25.57 (24.16, 27.95)	26.50 (21.94, 28.22)
BMI, N (%)			
Normal (18.5–24.9)	15 (38)	4 (33)	11 (39)
Overweight (25–29.9)	18 (45)	7 (58)	11 (39)
Obese (≥30)	7 (18)	1 (8)	6 (21)
Time since surgery, months (median, LQ, UQ)	121 (110, 148)	130 (109, 152)	120 (112, 133)
Ipsilateral breast surgery, N (%)			
Wide local excision	19 (46)	6 (50)	14 (50)
Mastectomy	12 (10)	2 (17)	8 (29)
Mastectomy + reconstruction	9 (17)	4 (33)	6 (21)
Contralateral surgery, N (%)			
Wide local excision	2 (5)	1 (8)	1 (4)
Mastectomy	2 (5)	0	2 (7)
Axillary surgery, N (%)			
Sentinel node biopsy/Sampling	4 (10)	0	4 (14)
Axillary lymph node dissection	36 (90)	12 (100)	24 (86)
Radiotherapy, N (%)			
Breast only	25 (63)	7 (58)	18 (64)
Breast and supra clavicular fossa	3 (8)	2 (17)	1 (4)
Breast and axilla	4 (10)	1 (8)	3 (11)
Breast, SCF and axilla	1 (3)	1 (8)	0
None	7 (18)	1 (8)	6 (21)
Dominant side treated N (%)			
Yes	14 (37)	6 (50)	8 (29)
Both sides	5 (12)	1 (8)	4 (14)
No	21 (51)	5 (42)	16 (57)
Chemotherapy, N (%)	25 (63)	9 (75)	16 (57)
Hormone treatment, N (%)	30 (76)	6 (50)	8 (29)
Cellulitis, N (%)	9 (22)	3 (25)	6 (21)
Patient reported onset of oedema, months after 1st axillary surgery (median, LQ, UQ)	7 (2, 12)	8 (6, 23)	5.5 (1.23, 12)
Patient reported onset of swelling, N (%)			
<3 months	8 (20)	2 (18)	6 (21)
3–6 months	2 (5)	1 (8)	1 (4)
>6 months	13 (33)	8 (67)	6 (21)
Cannot remember	1 (3)	1 (8)	0
Never developed lymphoedema	15 (38)	0	15 (54)
Reported trigger, N (%)	17 (43)	8 (67)	9 (32)
Received lymphoedema treatment, N (%)	20 (50)	11 (92)	9 (32)
Duration of swelling, months (median, LQ, UQ)*	102.5 (9.9, 129)	118 (96, 134)	54 (10.5, 108)

* No significant differences between groups except for duration of swelling, *p* *=* *0.025*

**Table 2:** Clinical characteristics of total group and according to presence of clinician-determined BCRL

Characteristic	Total group (N = 40)	Lymphoedema present (N = 12)	No current swelling (N = 28)	*P*
Clinical symptoms present, N (%)				
Decreased vein visibility	5 (13)	5 (42)	0	
Increased skin/subcutaneous thickness	4 (10)	4 (33)	0	
Fullness of tissues	15 (38)	12 (100)	3 (11)	
Pitting oedema	2 (5)	2 (17)	0	
Patient reported symptoms, N (%)s				
Pain	9 (23)	3 (25)	6 (21)	
Ache	5 (13)	3 (25)	2 (7)	
Heaviness	8 (20)	4 (33)	4 (14)	
ELV, ml (median, LQ, UQ)	1.5 (−63.5, 144)	120 (71.75, 171.75)	−49 (−97, 105.5)	*0.001*
% ELV	0.07 (−3.14, 5.71)	4.81 (2.65, 8.51)	−2.36 (−4.65, 3.47)	*0.001*
Limb volume difference (N = 39), N (%)				
<5 % ELV	27 (69)	6 (50)	21 (78)	
≥5 < 10 % ELV	10 (26)	4 (33)	6 (22)	
≥10 % ELV	2 (5)	2 (17)	*1 pt not measured	
Overall quality of life (LQ21 score) (best = 10, worst = 0)	8 (7.75, 9)	9 (8, 9)	8 (7, 9)	*0.028*
Physical domain (LQ F) (best = 10, worst = 40)	11 (10, 14)	12.5 (10.25, 14)	11 (10, 13)	>0*.05*
Appearance/body image domain (LQ A) (best = 5, worst = 20)	5 (5, 6)	6.5 (5.25, 7.75)	5 (5, 5)	*0.028*
Symptoms domain (LQ S) (best = 6, worst = 24)	8.5 (7, 11)	10 (7, 12)	8 (7.25, 11.75)	>0*.05*
Mood domain (LQ E) (best = 6, worst = 24)	10 (7, 11)	8 (7, 10)	10 (7.25, 11.75)	>0*.05*
QD score (best = 0, worst = 99)	15.91 (5.11, 22.73)	18.18 (7.39, 22.73)	12.5 (4.55, 24.43)	>0*.05*

### Lymphoedema prevalence

Nine women (23 %) had some degree of clinician-determined arm lymphoedema, which was also self-reported (see Table 3); four women (10 %) reported BCRL developed prior to attending the class. Only two women (5 %) reached the common diagnostic threshold of ≥10 % ELV, both of whom developed BCRL since attending the class. A further three women (8 %) had lymphoedema limited to the hand, also self-reported; thus the overall clinician-determined prevalence of BCRL was 30 %. Another three women self-reported current lymphoedema which was judged by the clinician not to be BCRL: two women had bilateral hand oedema likely due to arthritis, and the third woman experienced only the sensation of swelling in an area of paraesthesia in her posterior upper arm.

**Table 3:** Level of agreement between clinically-determined lymphoedema and other methods

Clinical assessment data, n = 40	Clinical assessment by researcher		Kappa
	Lymphoedema present n = 12	No current lymphoedema n = 28	
≥10 % ELV (whole arm)	2	0	0.217, *p* = *0.029*
≥5 % ELV (whole arm)	6	6	0.278, *p* = *0.083*
≥200 ml difference between arms	1	0	
Patient self-report, n = 40			
Current arm swelling	9^a^	1^b^	0.833, *p* = *0.000*
Current swelling limited to hand	3	2	
Swelling resolved	0	12	
Never swollen	0	13	

^a^Four women developed BCRL prior to attending the class

^b^Developed BCRL prior to attending the class

A further twelve women (30 %) without any current symptoms reported previous experience of lymphoedema affecting the hand, arm, axilla or breast; it is not known whether symptoms pre or post-dated attendance at the class. Eight women received lymphoedema treatment which included hosiery; five women reported full resolution achieved within 1 month. Three of the four women who underwent sentinel node biopsy experienced transient oedema, which they did not consider to be lymphoedema as it spontaneously resolved within 1 month.

Of the six women (13 %) who completed questionnaires but did not attend the clinical assessment, three had reported current swelling which could not be verified, two reported swelling now resolved, and one had never experienced swelling.

### Comparing diagnostic methods

The diagnostic method of ≥10 % ELV was 100 % specific (see Table 3) but had low sensitivity (17 %), identifying only two of the nine women with clinician-determined arm lymphoedema (k = 0.217, *p* = 0.029). There was a substantial level of agreement between clinician-determined swelling and patient self-report of current swelling (k = 0.833, *p* = 0.000, see Table 3).

### Evaluating the “reducing your risk of lymphoedema” class and exercise programme

All 46 women (100 %) responding to the questionnaire found the class beneficial. They particularly valued access to a knowledgeable health care professional, evidence-based information, specific advice regarding aspects of risk reduction and how to manage BCRL should it develop, the opportunity to share knowledge and experiences with other women in the same situation. They firmly believed their increased knowledge helped to prevent or minimise problems, supported their return to active daily life, and provided a strategy for self-management of risk and any subsequent BCRL. Only five women (11 %) reported both benefit from increased knowledge and alarm from heightened awareness. Forty-five women (98 %) recommended similar sessions and information be made available to everyone undergoing breast cancer treatment; for example:

“I hope that lymphoedema is taken more seriously by the medical establishment and that information & classes etc. now form part of a standard & holistic treatment plan” (ID38).

Most women (76 %) suggested the class should ideally be offered post-operatively; 25 women (54 %) suggested around 1 month and 11 women (24 %) suggested several months following surgery. Only 9 women (20 %) suggested it was best offered preoperatively, although three women thought offering both pre and postoperative classes was preferable. Several women suggested individual preference would affect the point at which both written information and class instruction would be most beneficial.

Thirty-seven women (80 %) reported benefit from being taught the exercise programme: 26 women (65 %) did the exercise programme for at least 1 month following the class, with 13 women (33 %) continuing the programme for at least 6 months (see Table 4). Some women reported still using the exercise programme whenever they became more aware of symptoms. Only one woman (2 %) stated the exercise programme was not helpful, although seven women (14 %) either could not remember the programme or recall whether it was beneficial. Twenty-nine women (63 %) indicated specific personal benefits, particularly increased confidence to know what they could safely do; the benefits include:

**Table 4:** Reported precautionary behaviour and activities following lymphoedema awareness class

Characteristics	Total group (N = 40)	Lymphoedema present (N = 12)	No swelling present	
			Swelling resolved (N = 13)	Never swollen (N = 15)
Lymphoedema precautions taken, N (%)	29 (73)	7 (58)	9 (69)	13 (87)
Frequency of exercise programme, N (%)				
Daily	25 (63)	9 (75)	9 (69)	7 (47)
Several times/wk	5 (12)	1 (8)	2 (15)	2 (13)
≤Once per week	4 (10)	1 (8)	1 (8)	2 (13)
Not done at all	1 (4)	0	0	1 (7)
Cannot remember	5 (12)	1 (8)	1 (8)	2 (13)
Duration of exercise programme, N (%)				
6+ months	13 (33)	6 (50)	3 (23)	4 (27)
3 < 6 months	7 (18)	1 (8)	3 (23)	3 (20)
1 < 3 months	6 (15)	2 (17)	3 (23)	1 (7)
1–4 weeks	1 (3)	0	0	1 (7)
Cannot remember	9 (23)	3 (25)	2 (15)	4 (27)
Missing	4 (10)	0	2 (15)	2 (13)
Current hobbies using affected hand/side	26 (65)	10 (83)	8 (62)	8 (53)
Current level of hand use				
Low	6 (15)	1 (8)	2 (15)	3 (20)
Moderate	27 (68)	10 (83)	9 (69)	8 (53)
High	7 (18)	1 (8)	2 (15)	4 (27)

“Relieve symptoms/aches and pains/tense muscles” (ID16,42,44),

“Eased movement of arms” (ID31),

“Gained strength in my arm” (ID40),

“Stretching affected arm in all directions” (ID37),

“I was able to see improvement” (ID46).

Many women wrote that they believed the knowledge they gained from the class helped to either prevent development of lymphoedema or resolve any swelling that did develop; for example:

“Due to the exercises & other activities I’ve listed lymphoedema is minimum” (ID8);

“Probably the most useful session I attended post surgery. Helped me avoid lymphoedema” (ID23).

The women also wrote about gaining from the class and exercise programme confidence to return to normal life and recommence a wide range of activities they might otherwise not have done. The exercise programme was used by some women as a bridge to other sports and exercise programmes; for example, following a return to Yoga or Pilates they gradually stopped doing the lymphoedema exercises. They reported a wide range of current active sports and hobbies, including squash, tennis, golf, gym, body-building, pole-walking, kayaking. Ten women (22 %) reported employment or a regular hobby requiring repetitive hand and arm movements, including pottery, glass-casting, mosaics, knitting, ceramics/clay-throwing, painting, drawing, sewing, woodcarving, upholstering furniture. Although the majority were very active, five women (11 %) volunteered that they had chosen to avoid or limit certain activities.

## Discussion

Our finding of 5 % BCRL prevalence, defined as ≥10 % ELV, is lower than might be expected when compared with other cross-sectional studies similarly defining BCRL (Disipio et al. 2013). When determined by clinician judgement, we found 23 % prevalence of arm lymphoedema, which increased to 30 % prevalence with inclusion of lymphoedema limited to the hand, with a median 4.8 % ELV. In the absence of a definitive reference method for determining BCRL, it is difficult to determine whether a method is under or over-diagnosing lymphoedema (Ward et al. 2015). However, we support the view that defining BCRL by limb volume difference underestimates the extent of the problem (Stanton et al. 2006; Armer and Stewart 2005; Disipio et al. 2013) and suggest that clinical judgement should be included to ensure appropriate diagnosis.

Clinician-judgement is highly interpretive. We wanted to capture all cases of lymphoedema so there was no threshold for inclusion of clinical symptoms. However, diagnostic parameters used by clinicians in other studies were often not specified thus making any meaningful comparison difficult (e.g. Hayes et al. 2008b; Ahmed 2006; Schmitz et al. 2010; Querci della Rovere et al. 2003). The challenge remains to quantify subjective diagnostic criteria to allow universal application and meaningful comparison of findings.

Reported incidence/prevalence of BCRL varies widely, influenced by the definition of lymphoedema used and characteristics of different assessment methods (Armer and Stewart 2005; Disipio et al. 2013; Hayes et al. 2005; O’Toole et al. 2013). Our study found that patient self-report correlated highly with clinician judgement of BCRL, which supports the view that the patient report should also be considered when determining the presence of BCRL (Armer and Stewart 2005; Paskett et al. 2012). A tool to aid self-report diagnosis may well have reduced the overestimation of self-reported lymphoedema (8 %) in our study (Armer et al. 2003; Norman et al. 2001). Other objective measures such as bioimpedance ratios and limb volume change (using a pre-surgery baseline) have been reported as beneficial in the early detection of lymphoedema (Perdomo et al. 2014) but, in the absence of a definitive reference method, their precision as a diagnostic method have yet to be determined.

There is a developing body of evidence to support the safety and benefit of exercise for women with and at risk of developing BCRL (Box et al. 2002; Kilbreath et al. 2012; Schmitz et al. 2010; Lacomba et al. 2010); this information this was not available at the time of the classes in 2000–2005. There is now recognition of the importance of conditioning the limb to cope with day-to-day activities and emphasising what can and should be done, rather than adopting a precautionary and restrictive approach to daily activity (Miller 1998; National Institute for Health and Clinical Excellence 2014; Schmitz et al. 2009, 2010; Jeffs et al. 2015). Several studies have shown benefit from exercise instruction combined with education about BCRL (Fu et al. 2014; Sisman et al. 2012). Group education about the importance of regular arm flexibility and strengthening exercises (in addition to general activity and maintaining range of shoulder motion) is a relatively low-cost way to inform women about positive approaches to daily living with or at risk of developing BCRL, rather than merely advising what they can safely do.

Lymphoedema awareness sessions have the potential to empower women to live well and live normally following breast cancer treatment, and provide guidance regarding pacing of exercise and activities. Our findings highlight the great value women place on this opportunity to gain evidence-based information and advice from a lymphoedema expert, with practical guidance to help them bridge the gap between post-operative exercises and various sporting activities and strenuous work roles. Women value expert information which increases their confidence to return to normal activity (Jeffs et al. 2015), but are frustrated by lack of information and poor quality or inaccurate information (Lee et al. 2010). In a study of 136 women following breast cancer treatment, those who received information about lymphoedema experienced fewer lymphoedema symptoms (Fu et al. 2010), although no conclusions could be drawn about the optimal format of information provision.

The study was not designed to identify causality, however, we can speculate regarding possible reasons for the low prevalence of BCRL defined by ELV. The women believed the education and exercise programme were very beneficial, particularly with regard to information about actions to minimise or prevent BCRL symptoms. Other factors will also have contributed to the BCRL outcome, including provision of lymphoedema treatment; the majority of those who had experienced BCRL had received some form of lymphoedema treatment.

The combination of patient report, clinical assessment and objective measurement to determine prevalence provides a strong level of confidence in the findings. We achieved a reasonable response rate to the questionnaire (60 %) and a high conversion rate (87 %) to the assessment phase; however, we do not know what happened to the 31 women (40 %) who did not return a completed questionnaire, nor were we able to confirm the self-report of the six women who completed questionnaires (part 1) but did not attend the assessment (part 2). The study population included women whose attendance at the class had been recommended by the local breast cancer service, and also women who had actively sought out the lymphoedema awareness class following breast cancer treatment elsewhere in London and the UK; we did not collect information about the hospital where the women received their cancer treatment.

Our findings have led us to conclude that clinician-judgement should be combined with objective measurement of ELV to avoid underestimation of BCRL, and to detect mild arm lymphoedema and also lymphoedema limited to the hand; in addition, patient self-report should be taken into consideration. Further research should be conducted to determine the protective and treatment benefits of providing group education from a lymphoedema expert and of teaching specific exercise; this is in addition to providing written information regarding lymphoedema care and general exercise.

## Abbreviations

BCRL: breast cancer-related upper limb lymphoedema
ELV: excess limb volume
GP: general practitioner
